# Exploring Dance Movement Data Using Sequence Alignment Methods

**DOI:** 10.1371/journal.pone.0132452

**Published:** 2015-07-16

**Authors:** Seyed Hossein Chavoshi, Bernard De Baets, Tijs Neutens, Guy De Tré, Nico Van de Weghe

**Affiliations:** 1 Department of Geography, Ghent University, Ghent, Belgium; 2 KERMIT, Department of Mathematical Modelling, Statistics and Bioinformatics, Ghent University, Ghent, Belgium; 3 Department of Telecommunications and Information Processing, Ghent University, Ghent, Belgium; Hellas, GREECE

## Abstract

Despite the abundance of research on knowledge discovery from moving object databases, only a limited number of studies have examined the interaction between moving point objects in space over time. This paper describes a novel approach for measuring similarity in the interaction between moving objects. The proposed approach consists of three steps. First, we transform movement data into sequences of successive qualitative relations based on the Qualitative Trajectory Calculus (QTC). Second, sequence alignment methods are applied to measure the similarity between movement sequences. Finally, movement sequences are grouped based on similarity by means of an agglomerative hierarchical clustering method. The applicability of this approach is tested using movement data from samba and tango dancers.

## Introduction

Technological advances in tracking and navigation systems make it possible to capture, efficiently and cost-effectively, the trajectories of a wide range of moving objects, including human beings [1, 2], animals [3–6], and vehicles [7, 8]. With access to an unprecedented wealth of accurate motion data, researchers today can apply pattern discovery techniques to moving object databases and generate knowledge in a large member of disciplines, including urban planning [9], event management [10, 11], crisis management [12], traffic [13], and tourism [14]. In addition to their usefulness for processing large-scale movement data sets, data mining and knowledge discovery techniques can also be applied to small-scale movement data sources. For example, movement patterns, such as walking, running, jumping, lifting, striking and swimming, can be investigated for various purposes. Investigating the movement of swimmers, for instance, might help coaches to analyse the performance of their swimmers [15]. Nonetheless, the specific techniques and methods chosen for extracting movement patterns from a data set depend on the context of the movement under examination. Among the wide range of research methodologies, similarity analysis has attracted considerable attention from many researchers. The similarity between two entities is measured as the cost of transforming one entity into another via a similarity measure [16]. In the context of movement, trajectories (i.e., representative paths that moving objects follow through space as a function of time) are typically considered to be the entities in similarity analysis of the dynamic behaviour of moving objects. In the existing research that has applied similarity analysis to the study of moving object trajectories, most studies have focused on the spatial dimension [17–20], whereas several studies have considered both spatial and temporal aspects [21–25]. However, despite extensive research in this field [26, 27], certain aspects of moving object trajectories have received only scant attention to date.

In this paper, instead of presenting a spatial or spatio-temporal similarity analysis of trajectories, we propose a framework in which the similarity measure is used to quantify similarity when pairs of moving objects interact with one another. We believe that a focus on the similarity in the interaction among moving object pairs may reveal more information on object movement than a sole focus on object trajectories.

To form the basis of the similarity analysis, a qualitative formalism appropriate for the representation of spatio-temporal human cognition is used to express the interactions between objects. To date, researchers have proposed several formalisms for the qualitative analysis of spatial and temporal phenomena. However, the existing work in this area has been limited to either spatial or temporal qualitative calculi [28–31], with only a few studies presenting an integrated, spatio-temporal treatment of object movements. One notable example of an integrative approach is the Qualitative Trajectory Calculus (QTC) [32]. QTC reduces the complexity of interacting, real-world, continuously disjoint moving objects by representing the interaction in terms of qualitative relationships [33]. By converting relative motion attributes (i.e., distance) into symbolic representations, QTC transforms quantitative data on movement (positional information) into qualitative data (QTC relations), resulting in a simplified representation of trajectory pairs. The practicality and appropriateness of QTC for analysing the interaction of moving objects have been successfully demonstrated via various applications [34–37].

In this paper, we cross-pollinate QTC with sequence alignment methods (SAMs) to identify similarities in the movement behaviour between pairs of interacting moving objects over time. Although SAMs have long been used in bioinformatics for the analysis of DNA strings [38], they have only recently been applied to the field of movement analysis [39]. In the current study, sequence alignment is used to assess the similarity between movement sequences of QTC relations for two reasons. First, SAMs allow us to visually distinguish movement patterns from sequences and extract insightful information from them. Second, the comparison of movement patterns using SAMs results in a quantitative measure of similarity between movement patterns. Finally, the results of a similarity analysis are used to cluster movement data into groups that share similar properties. The usefulness of our approach will be demonstrated in an empirical case study in which sequence alignment is used to examine the movement patterns of different parts of the body of samba and tango dance performers.

The remainder of this paper is organised as follows. Section 2 provides a brief review of the background and basics of QTC and SAMs, Section 3 presents a description of the data set used in this paper. Section 4 presents the methodology that is applied in this research. Section 5 discusses the proposed method, compares it with related approaches and identifies strengths and open problems. Finally, Section 6 presents our concluding remarks and outlines the directions for future work.

## Background

### The Qualitative Trajectory Calculus (QTC)

**Fig 1:**
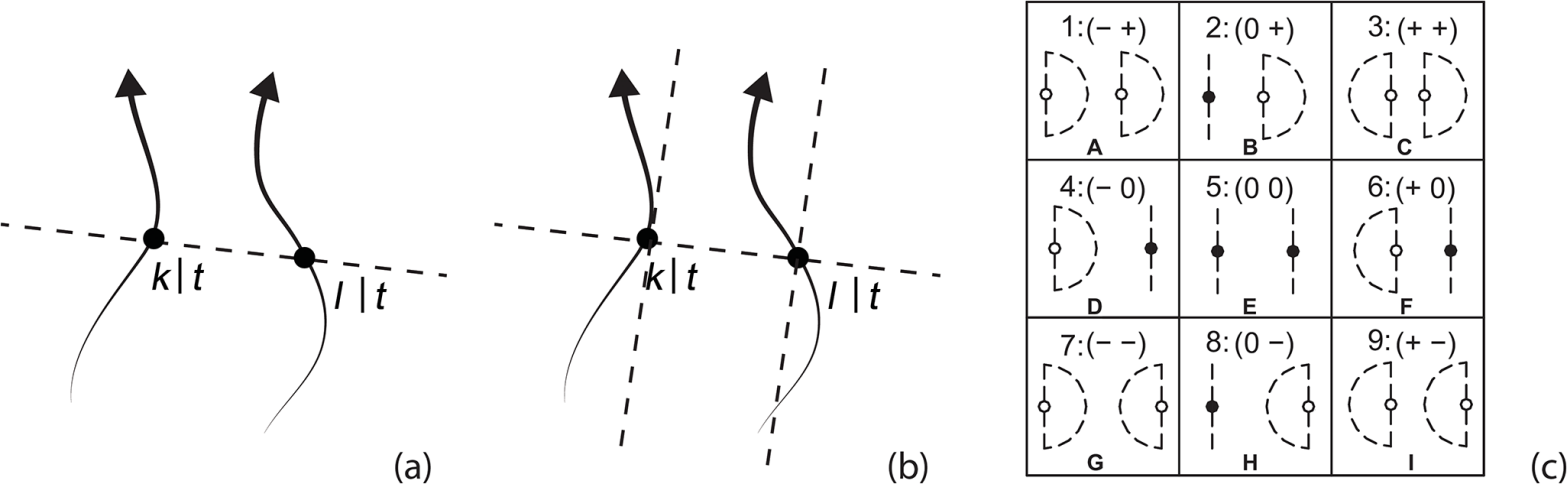
Two MPOs, *k* and *l*, and their trajectories are used to form QTC_B_ base relations. The frame of spatial reference is illustrated by the dashed line. (a), QTC_**C**_ setting (b). The frame of spatial reference is illustrated by the dashed line. Nine QTC_**B**_ base relations (c).

QTC was introduced by Van de Weghe [32] as a qualitative calculus to represent and reason about moving objects. It expresses the spatio-temporal relationship between two disjoint moving point objects (MPO_S_). Different types of QTC have been developed, namely QTC_B_ (QTC-Basic) [40], QTC_C_ (QTC-Double Cross) [41], QTC_N_ (QTC-Network) [42], and QTC_S_ (QTC-Shape) [43]. This paper will focus on QTC_B_ and QTC_C_. In QTC_B_, qualitative relations are defined based on the Euclidean distance between two disjoint MPOs at each time stamp of movement (Fig 1A), while QTC_C_ relations are determined based on three reference lines forming a so-called double cross between two disjoint MPOs (Fig 1B). In the remainder of this section, we will briefly introduce the basic concepts of QTC_B_ and QTC_C._

#### QTC_B_ (QTC-Basic)

QTC_B_ provides a qualitative representation of the two-dimensional movement of a pair of MPOs. Binary relations between two MPOs are evaluated based on the Euclidean distance [32]. QTC_B_ relations are constructed from the following relationships [36]:

Assume: MPOs *k* and *l*, and time point *t*

*k*|*t* denotes the position of *k* at *t*
*l*|*t* denotes the position of *l* at *t*
*d*(*u*, *v*) denotes the Euclidean distance between two positions *u* and *v*
A Movement of *k* with respect to *l* at *t* (distance constraint):
  -: *k* is moving towards *l*:

$$\begin{array}{l} \exists t_{1}(t_{1}<t\wedge \forall t^{-}(t_{1}<t^{-}<t\to d(k|t^{-},l|t)>d(k|t,l|t)))\wedge \\ \exists t_{2}(t<t_{2}\wedge \forall t^{+}(t<t^{+}<t_{2}\to d(k|t,l|t)>d(k|t^{+},l|t))) \end{array}$$

  +: *k* is moving away from *l*:∃t_2_(t ≺ t_2_ ∧ ∀t^+^(t ≺ t^+^ ≺ t_2_ → d(k|t, l|t) < d(k|t^+^, l|t)))

$$\begin{array}{l} \exists t_{1}(t_{1}<t\wedge \forall t^{-}(t_{1}<t^{-}<t\to d(k|t^{-},l|t)<d(k|t,l|t)))\wedge \\ \exists t_{2}(t<t_{2}\wedge \forall t^{+}(t<t^{+}<t_{2}\to d(k|t,l|t)<d(k|t^{+},l|t))) \end{array}$$

  0: *k* is stable with respect to *l* (all other cases)
B Movement of *l* with respect to *k* at *t* (distance constraint), can be described as in *A* with *k* and *l* interchanged, and hence:
  −: *l* is moving towards *k*
  +: *l* is moving away from *k*
  0: *l* is stable with respect to *k* (all other cases)

In QTC_B_, the distance constraints between two MPOs are denoted as *A* and *B*. Accordingly, the (*A B*)_B_ relationship syntax is used to represent the relation between two MPOs. In total, there are 9 (3^2^) base relations for QTC_B_ (Fig 1C). For example, the QTC_B_ relation (+ +) indicates that the two objects are moving away from each other.

#### QTC_C_ (QTC-Double Cross)

An important difference between QTC_B_ and QTC_C_ is that, in addition to the Euclidean distance, the direction of movement of MPOs with respect to the reference line (*RL*), the straight connection line between both MPOs (Fig 1B), is considered in the two-dimensional space. In other words, in addition to the towards / away from dichotomy of QTC_B_, QTC_C_ employs the left / right dichotomy. QTC_C_ relations are constructed from the following relationships [36]:

Assume: MPOs *k* and *l*, and time point *t*

*RL* ^*t*^ denotes the reference line through *k*|*t* and *l*|*t*

C Movement of *k* with respect to *RL* ^*t*^ at *t* (side constraint):
  –: *k* is moving to the left side of *RL* ^*t*^
  +: *k* is moving to the right side of *RL* ^*t*^
  0: *k* is moving along *RL* ^*t*^ (all other cases)
D Movement of *l* with respect to *RL* ^*t*^ at *t* (side constraint), can be described as in C with *k* and *l* interchanged, and hence:
  –: *l* is moving to the left side of *RL* ^*t*^
  +: *l* is moving to the right side of *RL* ^*t*^
  0: *l* is moving along *RL* ^*t*^ (all other cases)

**Fig 2:**
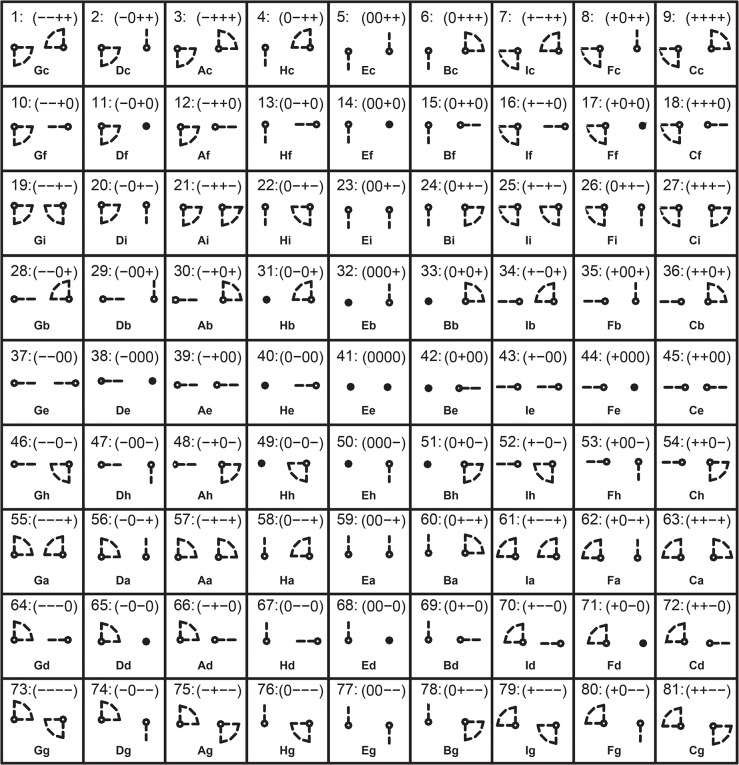
81 QTC_C_ base relations.

In QTC_C_, the *towards*/*away from* and *left*/*right* distinctions have been defined as notations *A*, *B*, *C*, and *D* respectively. Accordingly, the (*A B C D*)_C_ relationship syntax has been proposed for the relation between two MPOs at each time stamp of the movement. In total, there are 81 (3^4^) base relations for QTC_C_ (Fig 2). QTC_C_ relations thus reveal more detail of movement between two MPOs.

### Trajectories and QTC Movement Sequences

**Fig 3:**
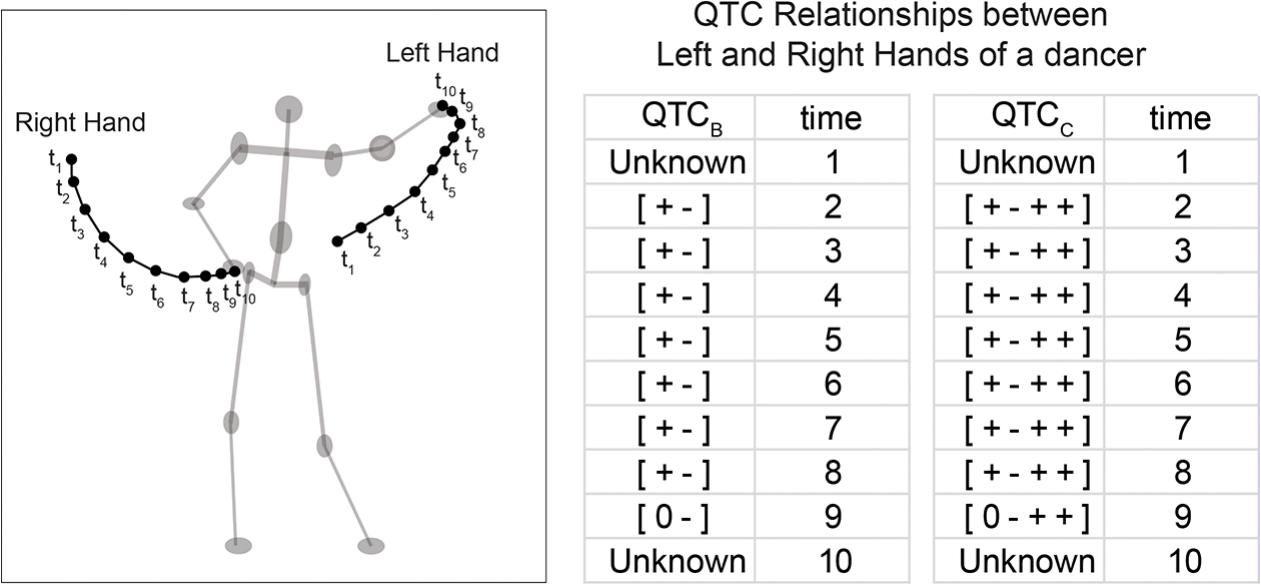
QTC_B_ and QTC_C_ relations of the movement of two hands of a dancer.

The trajectory of an MPO comprises a set of observations through space and time [44, 45]. A trajectory represents the movement of an individual MPO. The interactions between two MPOs during a time interval of movement can be expressed in the form of a QTC movement sequence– a chronological sequence of consecutive transitions between QTC relations. Fig 3 illustrates the movement of a pair of MPOs (i.e., hands of a dancer) during a 10-second interval with its QTC_B_ and QTC_C_ relations at each time stamp.

### Sequence Alignment Methods

Sequence alignment methods (SAMs) have played an important role in many research fields. In the early 1980s, biochemists began to use sequence alignment to analyse DNA sequences [39]. Later, social scientists, such as the sociologist Abbott [46], have applied sequence alignment to the analysis of career patterns. More recently, sequence alignment methods have been used in fields including transportation [47–49], cartography [50], tourism [51], and crowd behaviour analysis [52], among others.

Sequence alignment is the process of aligning two or more character sequences based on a set of conventional operations. Specifically, dynamic programming algorithms are used to equate sequences with the goal of maximising a similarity measure or minimising a distance measure between them [48, 53]. Two of the most widely used SAMs are pairwise alignment and multiple alignment. Pairwise alignment is the comparison of two sequences, whereas multiple alignment is the comparison of more than two sequences. Pairwise alignment and multiple alignment both operate on the basis of two primary types of algorithms: (i) global alignment and (ii) local alignment. Global alignment forces the alignment to span the entire length of all sequences, whereas local alignments identifies regions of similarity in long sequences (for a detailed explanation, see, e.g., [54]).

Pairwise alignment equates two sequences using four conventional operations: *identity*, *substitution*, *insertion* and *deletion*. Based on the scope of the research, each operation is associated with a cost or penalty that is defined *a priori*. The entire set of pairwise substitution scores is gathered in a scoring matrix.

In our case studies, we describe how SAMs can be used to align QTC movement sequences derived from the way in which dancers move different parts of their bodies and analyse these sequences based on the resulting similarities. In addition to the visual analysis of aligned QTC movement sequences, we present an objective assessment regarding how well dancers follow the instructions given by an instructor. In other words, our goal is to identify the aspects of students/beginners’ performances in which movement patterns of the dancers matched or deviated from the instructor’s movements.

## Data

The raw data used in this study were recorded using the MoCap (MotionCapture) system owned by the Department of Musicology at Ghent University. MoCap is a movement retrieval technique that records the position of objects over time by means of reflective markers attached to these objects in combination with infrared cameras. It is used in a wide range of research fields. For example, MoCap has been used in sports sciences to capture the movement of athletes as part of rehabilitation, physical education and practice [55, 56]. In the medical sciences, physiotherapists, orthopaedists and neurologists may examine MoCap measurements of human gait in conjunction with biomechanical modelling to evaluate a patient’s status and develop plans for treatment and rehabilitation [57]. Here, we examine some of the basic movements in two different types of dances, namely samba and tango.

Samba is a rhythmical dance. Characterising the conformity of samba dance movements is highly meaningful given that samba is a dance that involves a group of dancers rather than a single one.

The movements of the three samba dancers’ heads, torsos, right and left hands, and right and left feet at each time stamp of a considered time interval of 3.64s (temporal granularity of 0.04s) were recorded in the following format: *t* (i.e., the time stamp of movement), *x*, *y*, and *z* (i.e., the local positional information in a three-dimensional space) of each captured body part. The recorded positions of the markers were transformed into coordinates using the torso of a dancer’s body as origin. Across 92 time units, many repetitive movements were observed from the performances of the teacher and the two students. Datasets and Videos of the movements analysed in this study are available (S1 Dataset and S1 Video).

Next to samba, we will consider an example of tango. Tango is a sensual ballroom dance usually performed by a couple, a man and a woman, expressing an element of romance in their synchronised movements. Basically, tango consists of pivots and steps of either partner. The moment a man opens his chest (i.e., dissociation), the woman will pivot and go in the direction where the man opened his chest (i.e., steps). A basic step (i.e., Caminada) of a couple of tango dancers is considered in this paper.

We recorded the movements of a couple of professional tango dancers and a couple of beginners. Although we captured 25 body parts for each tango dancer at each time stamp of movement (i.e., 0.01 s), for the sake of simplicity, we only consider the most important body parts during a tango performance, namely shoulders, hips and feet. Movements of the professional tango dancers were registered with a calibrated MoCap system, while capturing devices were not adjusted well during recording the movements of amateurs intentionally. Consequently, there exist some errors and missing points in the dataset of the beginners. Clearly, the beginners performed less on time than the professionals. Synchronicity in performance is the factor that most effectively draws people’s attention. Not only is synchronicity important to dancing, it may be used as a qualification measure for other types of movements such as synchronised swimming—a hybrid form of swimming, dance and gymnastics—that consists of swimmers performing a synchronised routine of complicated moves in the water, accompanied by music.

### Ethics Statement

The data used in this study were obtained from the movements of three samba dancers and two couples of tango dancers. Participants were three volunteer samba dancers and two couples of tango dancers whose movements were captured at the IPEM research group, Department of Musicology of the Ghent University. This dataset contains *X*, *Y*, and *Z* coordinates of the body parts of dancers. There is no identifying information associated with any of the individuals, and thus this research does not constitute any risk to make the data available for public. In addition, the dancers have given verbal informed consent to use the data for publication purposes. The oral consents are documented by Dr. Luiz Naveda and Tim Vermeulen who was in charge of obtaining data from dancers (http://www.ipem.ugent.be/user/19).

## Methodology

In contrast to existing methods of classification, in this study, we measure the similarity of interactions between pairs of MPOs. In other words, instead of comparing individual trajectories, we compare pairs of trajectories for similarity. We follow three major steps. First, raw trajectories of interacting MPOs from location-aware technologies are converted into qualitative relations (QTC_B_ and QTC_C_). Second, sequences of the qualitative relationships are aligned for the interpretation of the movement patterns of MPOs. Finally, the results of the alignment are used to evaluate the dance performances. Each step is discussed in depth below.

### Step 1: Converting raw trajectories of MPOs into qualitative relations

#### Case 1: Samba dance

**Fig 4:**
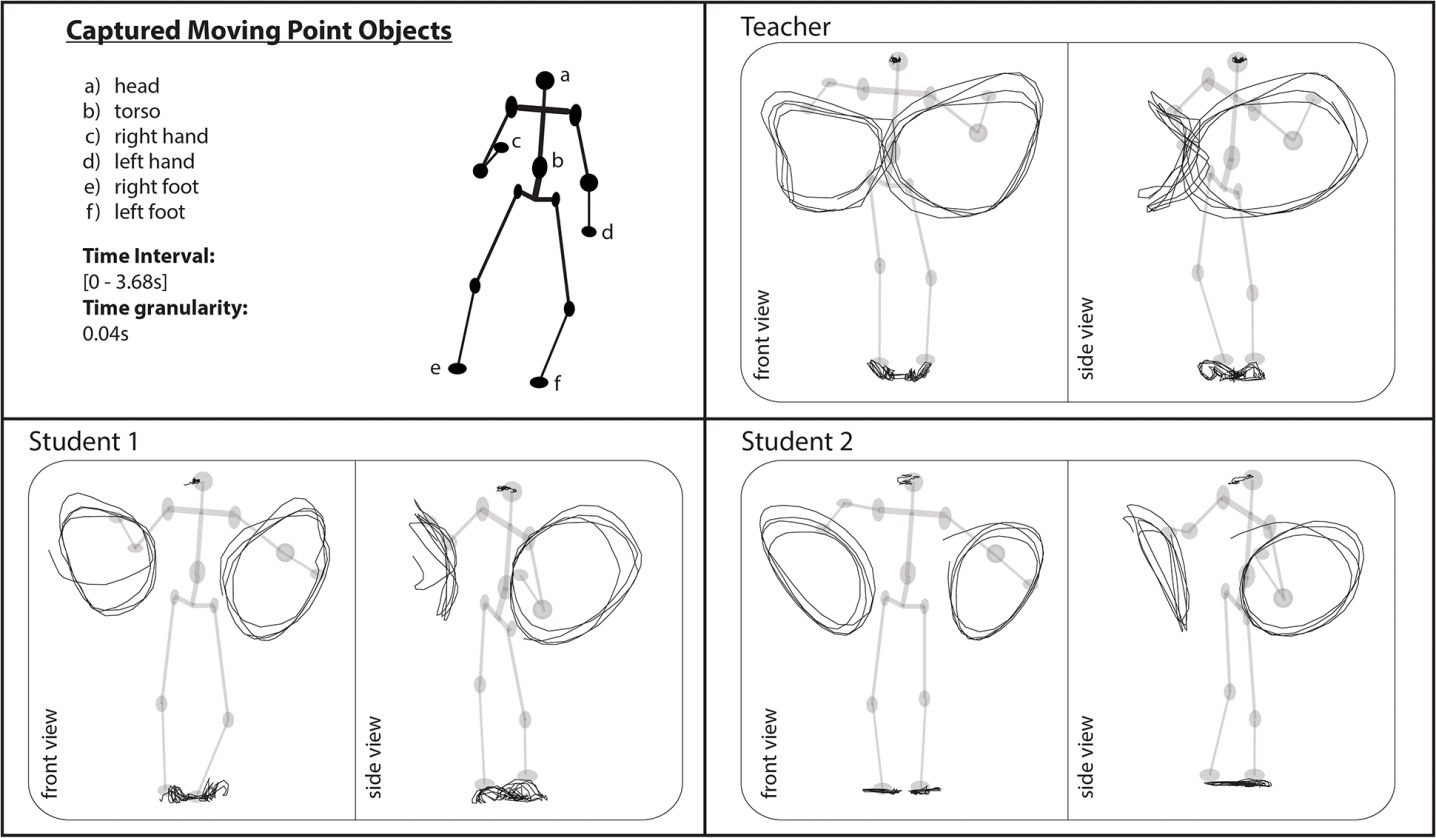
Derived trajectories of the movements of different parts of the body of samba dancers using the MoCap system.

In the first step, the relationships between different parts of the body of the three samba dancers are described in terms of QTC_B_ and QTC_C_ relations. For example, Fig 4 presents the movement of the dancers’ heads, torsos, right and left hands, and right and left feet in a given time interval from both the front view and the side view. The trajectories of the teacher’s body parts to those of the students reveal several subtle differences. For example, from the front views displayed in Fig 4, we can observe that the space used by students to move their hands was quite different compared to that of their teacher.

**Fig 5:**
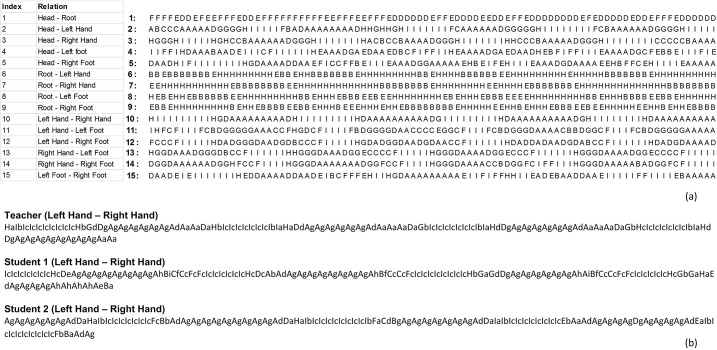
All QTC_B_ movement sequences of the teacher during 3.64s of movement (a), QTC_C_ movement sequences of the hands of the teacher, student 1, and student 2.

Next, for simplicity, QTC_B_ and QTC_C_ relations were transcoded into single-character and two-character sequences, respectively. The corresponding character code for each base relation in QTC_B_ and QTC_C_ is presented in Figs 1B and 2, respectively (below each representation). Fig 5A presents the entire set of transcoded sequences of QTC_B_ relations between the different limbs of the teacher (i.e., *n*(*n* − 1)/2 with *n* the number of body parts) in a movement lasting 3.64s (temporal granularity of 0.04 s).

In the case of QTC_C_, each code has two characters in which the first character refers to the first two symbols of the QTC_C_ relation (distance constraints) and the second character to the last two symbols of the QTC_C_ relation (direction constraints). For example, QTC_C_ relation (0 + + +) is replaced by the code *Bc*. In order to better detect transitions from one QTC_C_ relation to another, the first character in each code is capitalized. Eventually, a QTC_C_ movement sequence shows interactions between a pair of MPOs during a time interval of movement. Fig 5B illustrates the QTC_C_ movement sequences of the hands of the samba dancers during a time interval lasting 3.64s. Note, in all cases, we use QTC information in 2 dimensional space

**Fig 6:**
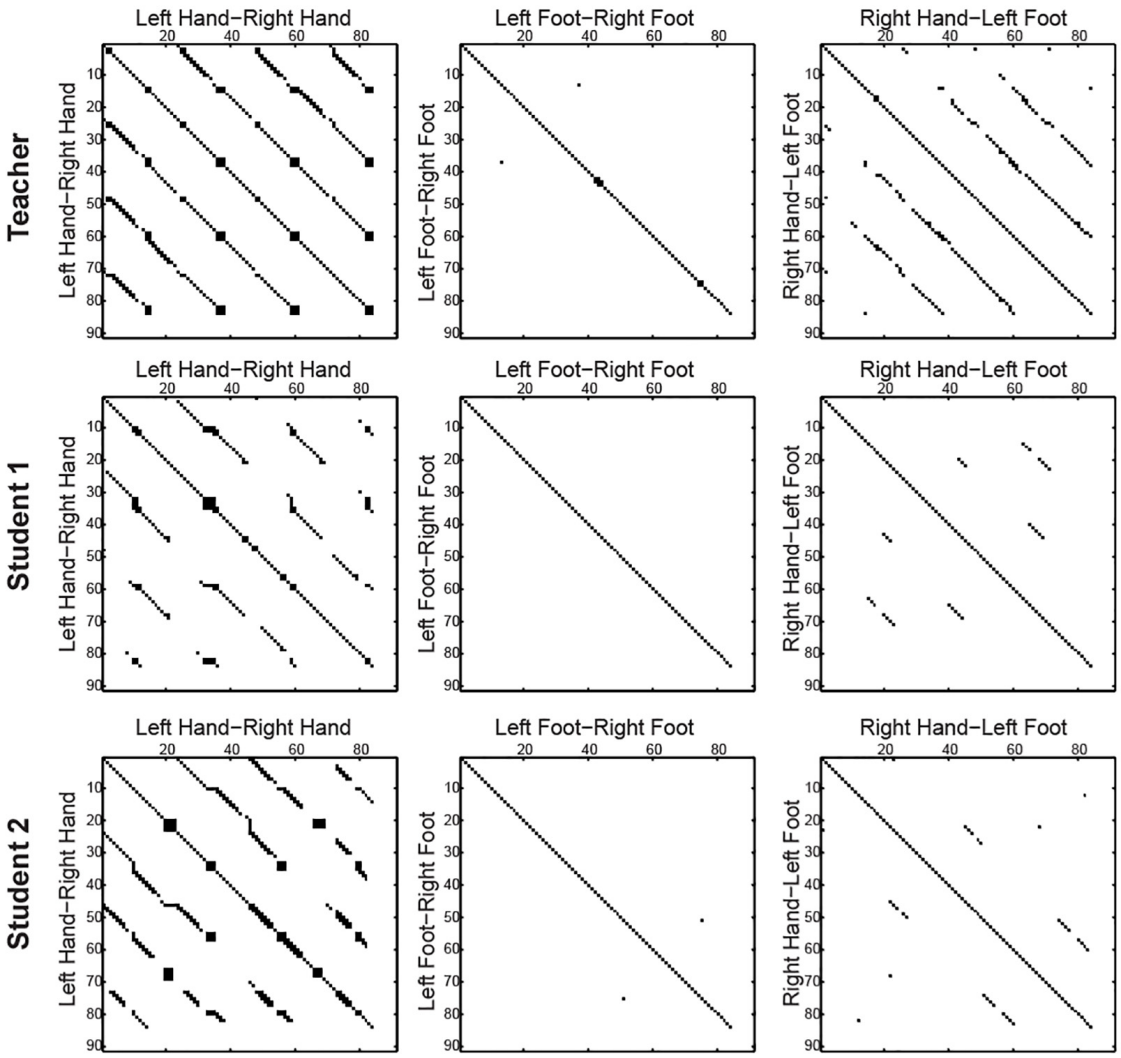
Dot plots of QTC_B_ movement sequences of *left hand—right hand*, *left foot—right foot*, and *right hand—left foot* for three samba dancers.

As stated earlier, samba dance is a dance with numerous periodic movement patterns, which can be discovered via an analysis of the QTC movement sequences of dancers. One way to visually recognise the periodicity in movement sequences is mapping sequences to dot plots. From a dot plot, certain sequence features (such as ‘repeats’) can be visually identified [58]. Dot plots are constructed using two sequences– one written along the top row and the second written along the leftmost column of a two-dimensional matrix. In a dot plot, each dot represents a point at which there is a match between the characters in the corresponding row and column. Thus, it is possible to identify a certain number of matches in a sequence in a search window defined *a priori*. Repetitiveness in a single sequence can be assessed by plotting a sequence against itself in a dot plot and sections that share similarities become visible in the form of lines off the main diagonal. Fig 6 comprise dot plots of the QTC_B_ movement sequences for three pairs of body parts (i.e., *left hand—right hand*, *left foot—right foot*, and *right hand—left foot*) for the teacher, student 1, and student 2. To derive the plots, we run a window spanning 10 characters along movement sequences in which 8 characters are matched. Many repetitive sequences of relative movements can be observed in the dot plots of *left hand—right hand* for all three samba dancers, whereas almost no repetition is observed in the QTC_B_ relations of *left foot—right foot* with a window of the same size. Regularity is more visible in the movement of hands than in those of feet. The neat straight lines in the *left hand—right hand* dot plot for the teacher indicate regular and perfect repetitions of the teacher’s movements over time. The lines in the dot plots for students 1 and 2 show various deviations, and are not as straight as those of the teacher. These deviations are caused by lag and lead times in the repetition of the same movements by the students. Based on these plots, we can roughly infer that the movements of student 1 and 2 are not as regular as the movements of the teacher. Next, we will further examine this irregularity in the students’ movements via sequence alignment and attempt to identify them automatically.

#### Case 2: Tango dance

In tango, the movement of couples is of interest. There exist many forms of tango. In this study, we consider the Argentina tango style in which couples follow a close embrace, a type of closed position where the leader and the follower stand facing each other chest-to-chest in full or partial body contact. The foundation of the Argentine Tango is like walking and is called Caminada. Compared to ordinary walking, the Caminada distinguishes itself on three aspects: more upright, in a narrower track and a bit like a prowling cat. In this paper, we study the fundamental step in tango dance, Caminada. A professional tango dancer aided us in obtaining a better understanding of the tango dance movements and recognizing the most important moments which can be taken as criteria to differentiate a good performance from a feeble one.

**Fig 7:**
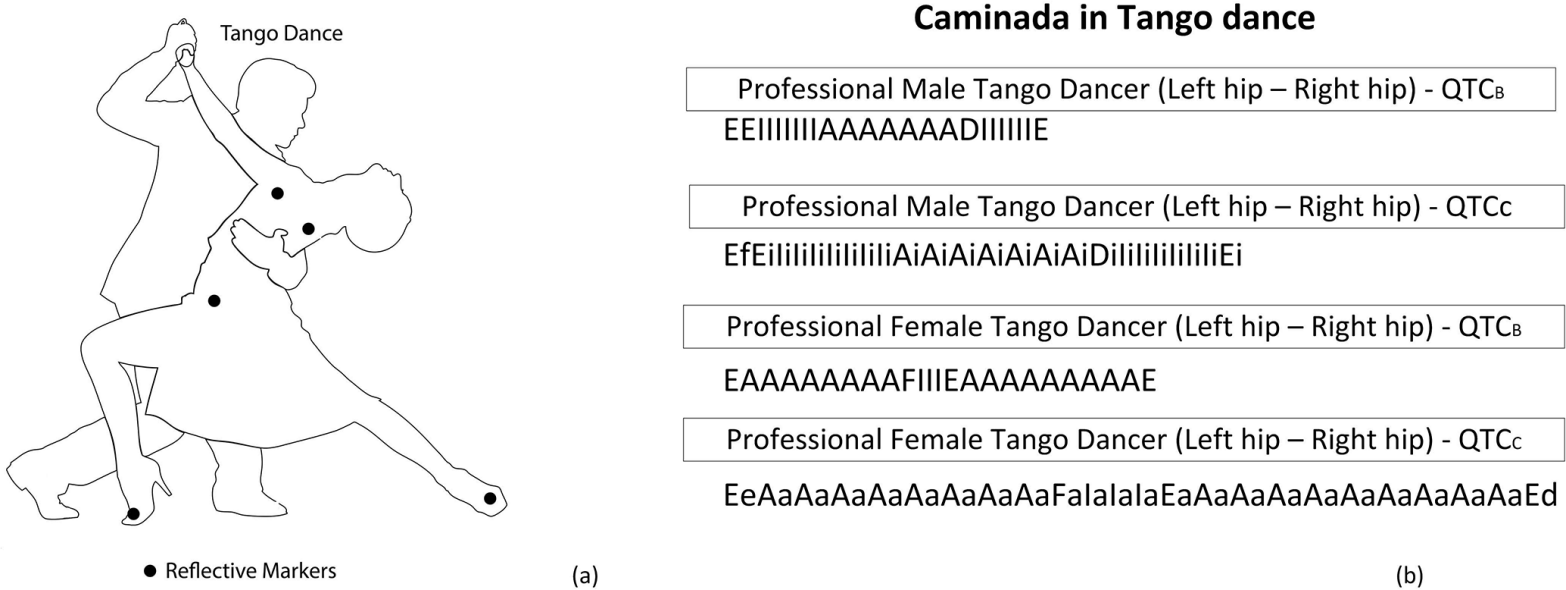
Selected reflective markers (i.e., shoulders, hips, and feet) (a), an example of QTC_B_ and QTC_C_ movement sequences (i.e., ships) of the couple of professional tango dancers (b).

The relative movements of the body parts of each tango dancer (i.e., shoulders, hips, and feet) are formalized by QTC_B_ and QTC_C_ relations. In Fig 7A, the selected reflective markers attached to the body parts of the tango dancer are illustrated. In Fig 7B, an example of important movement sequences of the hips of a couple of professional tango dancers is given. In this study, we only consider the relative movements of body parts of each dancer individually. However, it would also be of interest to examine the relative movement of one body part of a dancer with respect to that of the partner.

### Step 2: Aligning QTC movement sequences

In the second step, we align the QTC movement sequences of different body parts of the dancers. Using SAMs, we determine the degree of similarity between the movements of dancers during their performance. Finally, we evaluate the overall performance of each dancer based on the similarities resulting from the alignments.

The main challenge is to optimally align the QTC movement sequences of the students/beginners with the movement sequences of the teacher/professionals. Sequence alignment is applied to identify the parts of the students/beginners’ performance that matches or mismatches the performance of the teacher/professionals. When the differences between the aligned QTC movement sequences of the teacher/professionals and the student/beginners are sufficiently small, we can conclude that the student/beginners have performed their movements very well on the basis of the teacher/professionals’ movements as the choreographic benchmarks. Clearly, not all movements of the student/beginners’ bodies comply with the benchmark. To visualise and analyse the (dis)similarity between the body movements of the students/beginners with respect to that of the teacher/professionals, we examine short time intervals of their performances. We deliberately keep the time intervals short to make it easier to recognise (dis)similarity in the movement sequences and study the basic movements of dancers.

As mentioned earlier, the alignment of two sequences is based on minimising the distance between them (using a pre-defined scoring matrix). Performing sequence alignment on two sequences yields: (i) the distance (or similarity) between two sequences and (ii) the best possible alignment of the two sequences, which is the alignment that minimises the overall distance between the two sequences.

Two different scoring matrices for QTC_B_ and QTC_C_ relations are defined based on the *conceptual distance* [59] of QTC relations. The conceptual distance is defined as a measure of closeness of two QTC relations by counting the number of changes in the symbols of the QTC representation (*A B*)_B_ and (*A B C D*)_C_ [59]. The smallest conceptual distance is zero (i.e., the distance between a QTC_B_/QTC_C_ relation and itself). The conceptual distance between ‘0’ and ‘+’ or ‘–’ is one. The conceptual distance between ‘–’ and ‘+’ equals two because direct transition is impossible [60]. The overall conceptual distances between two QTC relations can then be calculated by summing up the conceptual distance over all relation symbols and rescaling it to the interval [0 10]. Therefore, a similarity score between two QTC_B_ relations can then be calculated as (10 – 2.5 * conceptual distance). For example, Table 1A presents the resulting QTC_B_ scoring matrix based on the transcoded QTC_B_ relations in Table 1B. An exact character match is assigned a similarity score of 10 (maximal similarity) and a total mismatch is given a similarity score of 0 (maximum conceptual distance). For instance, the conceptual distance between the two QTC_B_ relations (– +) (i.e., character A) and (– 0) (i.e., character D) is equal to one. For every conceptual distance unit, the similarity score decreases by 2.5 units from the maximal similarity score of 10. Therefore, the similarity score between A and D is equal to 7.5.

**Table 1:** Sequence alignment scoring matrix for QTC_B_ relations.

A										B	
Similarity Matrix	A	B	C	D	E	F	G	H	I	QTC_B_ Relations	Code
**A**	10	7.5	5	7.5	5	2.5	5	2.5	0	(- +)	**A**
**B**	7.5	10	7.5	5	7.5	5	2.5	5	2.5	(0 +)	**B**
**C**	5	7.5	10	2.5	5	7.5	0	2.5	5	(+ +)	**C**
**D**	7.5	5	2.5	10	7.5	5	7.5	5	2.5	(- 0)	**D**
**E**	5	7.5	5	7.5	10	7.5	5	7.5	5	(0 0)	**E**
**F**	2.5	5	7.5	5	7.5	10	2.5	5	7.5	(+ 0)	**F**
**G**	5	2.5	0	7.5	5	2.5	10	7.5	5	(—)	**G**
**H**	2.5	5	2.5	5	7.5	5	7.5	10	7.5	(0 -)	**H**
**I**	0	2.5	5	2.5	5	7.5	5	7.5	10	(+-)	**I**

Analogously, a scoring matrix based on the concept of conceptual distance is introduced for QTC_C_ movement sequences. This is a well-defined matrix in the same proportion as the scoring matrix for QTC_B_ movement sequences. It is an 81x81 symmetrical matrix in which each cell indicates the conceptual distance between two QTC_C_ relations. The maximum conceptual distance is 4 for QTC_B_ relations, while it is equal to 8 for QTC_C_ relations as there are four symbols in QTC_C_ relations.

Two parameters that need to be set in the process of sequence alignment are gap opening and gap extension. In this paper, *insertion/deletion* penalties for gap opening and for gap extensions are—5 and—3, respectively. In SAMs, dynamic programming algorithms are used in the search for optimal alignment to either maximise a similarity measure or minimise a distance measure based on the predefined scoring matrix [48].

#### Case 1: Samba dance

Each samba dancer has 15 QTC movement sequences representing 15 interacting pairs of body parts. Because the dancer’s torso is used as a reference point for the movement of other body parts, movement sequences involving torso (i.e., root) are not considered in the alignment process. Using the specified similarity scores and penalties, a multiple alignment of QTC movement sequences is generated with the ClustalTXY software package [48] based on the progressive alignment procedure. At a given time, three corresponding QTC movement sequences (i.e., of the teacher and the two students) are aligned followed by a multiple alignment using a global alignment [61].

**Fig 8:**
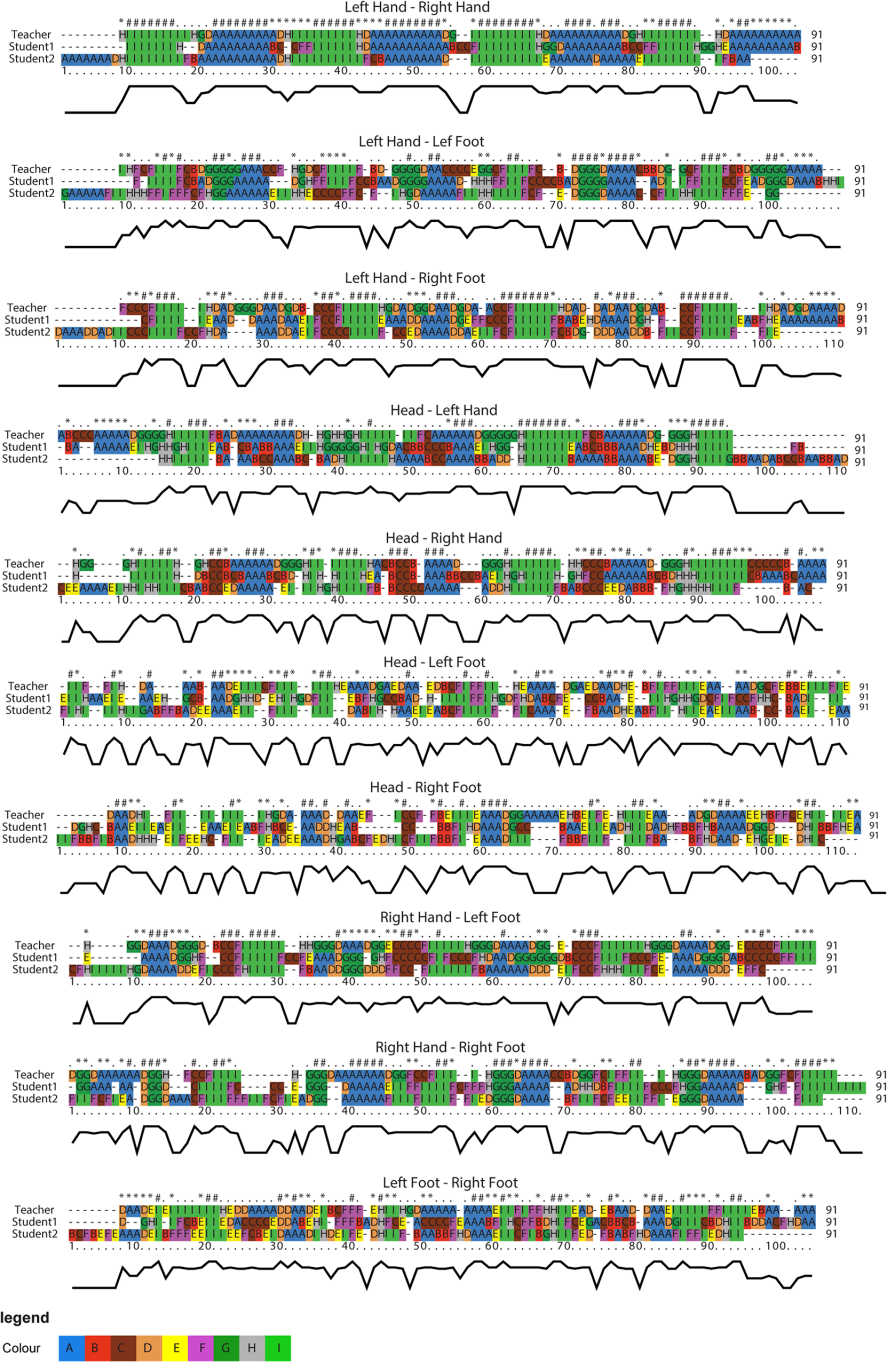
Multiple alignment of QTC_B_ movement sequences of dancers’ body movements.

Fig 8 presents the results of the alignment of QTC_B_ movement sequences. For clarity, the characters (i.e., transcoded QTC_B_ relations) have been colour-coded. The row above the aligned sequences is used to mark strongly conserved positions. Four characters are used to indicate the degree of matches: '#' indicates positions that are 80%-100% identical, '*' indicates positions that are 60% -80% identical, ':' indicates positions that are 40% -60% identical, '.' indicates positions that are 20% -40% identical. The curve below the movement sequences represents the rate of changes in the match and mismatch of characters at each time stamp of movement after sequence alignment. Less fluctuation in curves with highly matched characters at each time stamp indicates more similarity between movement sequences. The results show the (lack of) regularity in dance movement patterns. For instance, the sequences representing the *left hand—right hand* relations exhibit periodicities in the dancers’ movements. This pattern can be observed from the succession of colours and attributed to the fact that dancers paid more attention to the movement of hands than to other parts of the body. Moreover, the relative movements of head and hands show more regularity than the relative movements of head and feet, suggesting that dancers were more successful in adjusting the movement of the upper part of their body relative to the lower part. From the sequences of *left foot—right foot* relations, it can be observed that the rate of change in movement patterns is rather high compared to those of the hands.

**Fig 9:**
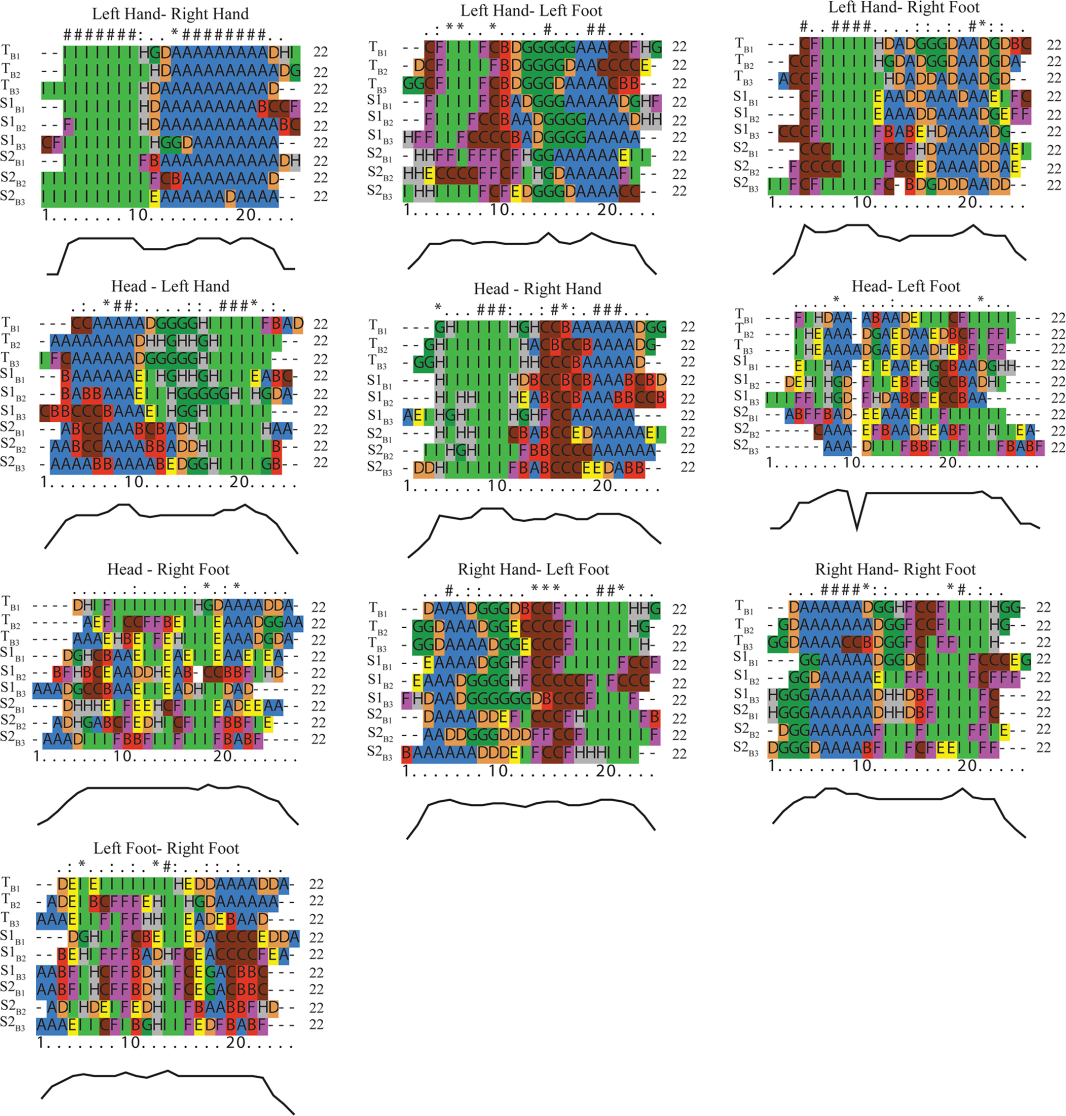
Multiple alignments of QTC_B_ movement sequences based on the beats of the music.

Using sequence alignment, repetitive movement patterns for each dancer can be individually assessed as smaller units of the entire performance. For this purpose, the rhythm in the music is used to mark the starting and ending points of the repetitive movement patterns. In our case, the entire performance lasts 91 time units and consists of 3 complete repetitive patterns that each last 22 time units. Aligning these repetitive movements allows us to examine the degree of similarity between the performances of dancers across successive beats. Fig 9 presents the results of aligning the movement sequences for each pair of body parts in relation to the musical beat.

**Fig 10:**
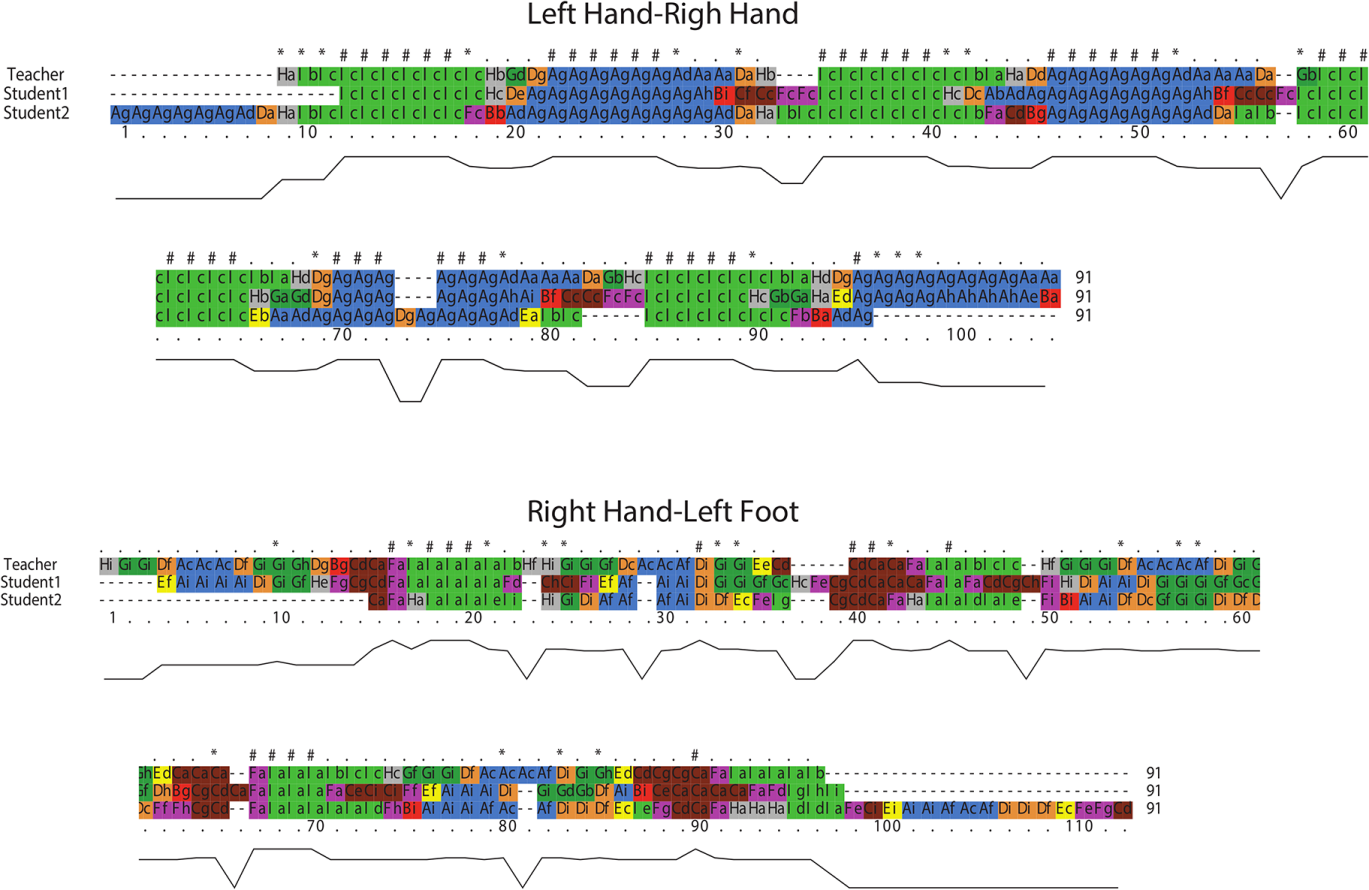
Multiple alignment of QTC_C_ movement sequences of *left hand*—*right hand* and *right hand*-*left foot* of samba dancers.

Up to now, we have relied only on QTC_B_ information that is based on Euclidean distance between MPOs and have thus disregarded the directional information of movement of MPOs. An important difference between QTC_B_ and QTC_C_ is that, in addition to the Euclidean distance, QTC_C_ takes into account directional information of movements as introduced earlier in this paper. This extra information can be used to achieve better insight into movement behaviour of MPOs and understand the processes behind movement patterns. As an example, Fig 10 demonstrates the aligned QTC_C_ movement sequences of (*right hand*—*left hand*) and (*right hand*—*left foot*) of students and teacher taking into account the QTC_C_ scoring matrix. To enable a visual exploration of movement sequences and improve the interpretability of the results, the characters have been colour-coded in a similar way as in Fig 8 with nine distinguishable colours. Each two characters in a QTC_C_ relation are assigned a unit colour taking the first character of each QTC_C_ relation to colour it. The QTC_C_ movement sequences embody more detailed information of movements. As expected, the movements of hands of the three dancers still have the best alignment with respect to other parts of the body.

#### Case 2: Tango dance

**Fig 11:**
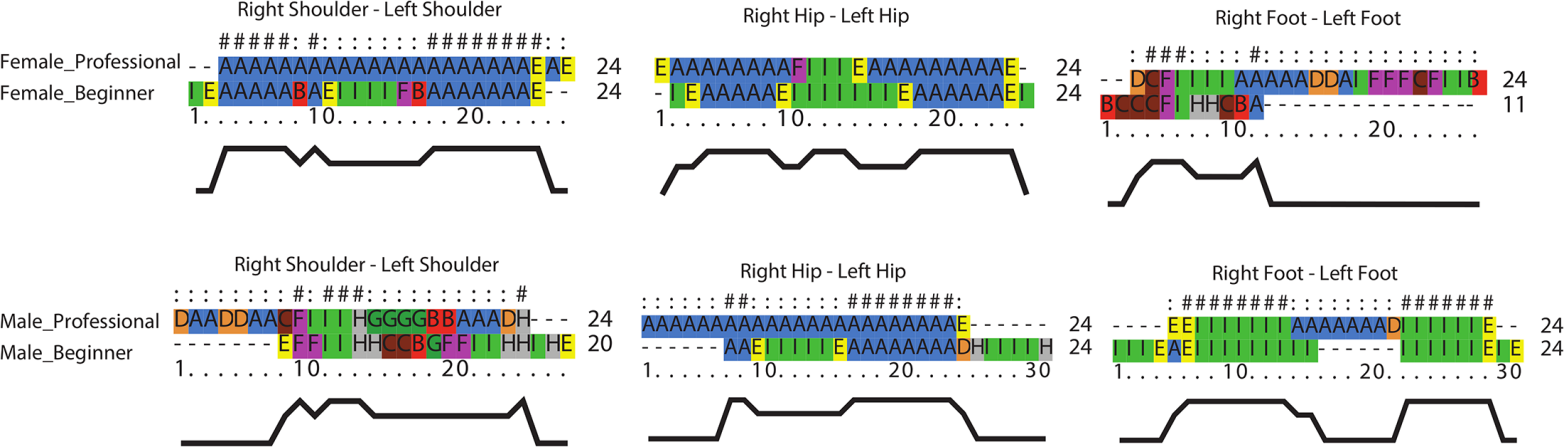
Pairwise alignment of QTC_B_ movement sequences for tango dancers.

In the tango case, we only consider three interacting pairs of body parts, namely shoulders, hips, and feet. Some of the results of aligning QTC_B_ movement sequences for tango case are presented in Fig 11. In this figure, one may observe which body parts of beginners moved in a similar/dissimilar way to that of the benchmark (i.e., professionals).

In Section 5, we will examine whether the proposed methodology can handle movement which may include gaps, noise, non-equidistant sampling intervals, and non-cyclical movement patterns and how robust the methodology would perform in the presence of uncertainty (e.g. measuring errors of positions in the movement data) and data gaps.

In addition to visually characterising the similarities/differences in movement patterns of dancers based on the rhythm of the music (i.e., Teacher beat 1 (T_B1_), Teacher beat 2 (T_B2_), Teacher beat 3 (T_B3_), Student 1 beat 1 (S1_B1_), Student 1 beat 2 (S1_B2_), Student 1 beat 3 (S1_B3_), Student 2 beat 1 (S2_B1_), Student 2 beat 2 (S2_B2_), and Student 2 beat 3 (S2_B3_)), we further present a numerical measure based on alignment scores, represented in the form of hierarchical clusters of movement patterns and histograms.

### Step 3: Overall evaluation of the performances

Clustering enables the detection of objects that share similar properties. Clustering is typically application dependent. In this paper, we attempt to cluster the dancers’ movements based on the relative motions of various body parts. We use a hierarchical clustering method to build a hierarchy of clusters (i.e., the movement sequences of the dancers). Based on the results of multiple alignments of QTC_B_ and QTC_C_ movement sequences, we evaluate the general performances of dancers.

#### Case 1: Samba dance

**Fig 12:**
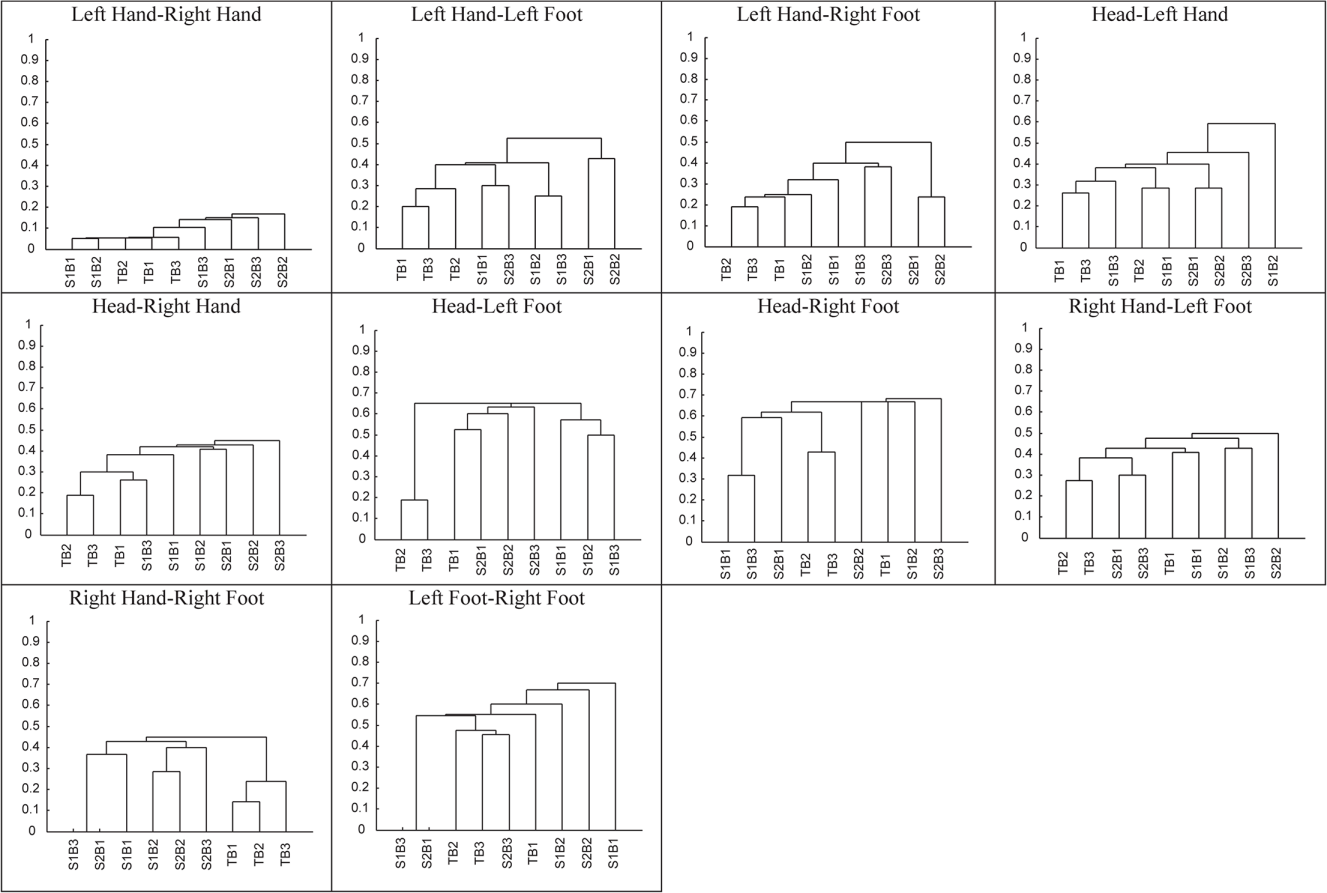
Dendrograms based on Fig 9.

For example, the results of the clustering of QTC_B_ movement sequences in samba case are represented in the form of dendrograms in Fig 12. A dendrogram supports the determination of a typology of different movement behaviours of dancers. The results of applying sequence alignment to real dance data suggest that certain movements were harder to follow by the students than other movements. Fig 12 shows the agglomerative hierarchical clustering in the form of dendrograms for the sequences as presented in Fig 9.

The height of the branch points shows the extent to which clusters differ from one another: the greater the height, the greater the difference. The value 0 represents the minimum distance after aligning the movement sequences, whereas 1 represents the maximum distance. As shown in the dendrograms, distances vary from one pair of body parts to another. In Fig 12, for example, the relative motion of the teacher’s hands did not differ significantly from that of the students, as demonstrated by the relatively small distance in the *left hand—right hand* dendrogram. In contrast, the *head—left foot* dendrogram shows a significant difference between the last two beats of the teacher and the other beats. Based on this method of alignment and clustering, we notice that the performance of student 1 is better than that of student 2. Furthermore, this method allows us to identify the pairs of student body parts that more closely resemble those of the teacher. These results can assist instructors in recognising the strengths and weaknesses in their students’ performance in the process of learning dance.

**Fig 13:**
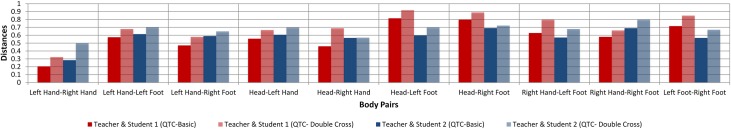
A sample assessment of the performances of the samba students.

To show the impact of each parameter (i.e., distance and direction) on the final results, a histogram is given in which the overall performances of students are compared to that of the teacher both for QTC_B_ and QTC_C_ information (Fig 13). In this histogram, more detailed information can be retrieved and interpreted. The result shows which body pairs of the samba students were moving correctly with respect to the teacher’s movements. For instance, as shown in Fig 13, student 1 could match the relative movements of his hands to those of the teacher, whereas student 2 succeeded to relatively move his feet in a manner highly consistent with that of his teacher. Moreover, although student 1 showed that he could control the relative distance between his head and right hand over time, he could not manage to control the relative directions of his movements in a manner similar to that of the teacher. The results of this approach are comparable with those in [62].

#### Case 2: Tango dance

**Fig 14:**
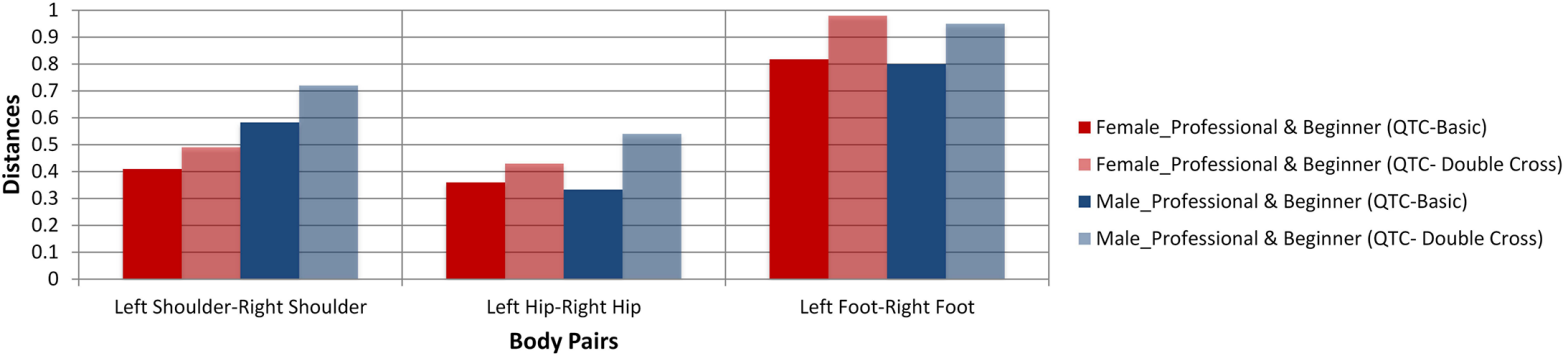
A sample assessment of the performances of the tango dancers.

In this subsection, we present the results of our comparison for the most complete recorded tango data. Fig 14 shows a histogram in which the overall performances of beginners are compared to those of professionals both for QTC_B_ and QTC_C_ information. The result shows that the male partner moved his shoulders better with respect to that of the female partner. Moreover, according to the results of alignments for movement sequences of hips, we notice that the male partner could not perform well enough in regard to keep right directions consistently.

## Discussion

Much progress has been made regarding the theories, methodologies, and applications for analysing, modelling, and interpreting movement data. Researchers have focused on different aspects in this area, including analysing the sequential aspects within the spatial and temporal dimensions of movement data (e.g., [39, 52, 63, 64]). For example, in [62], key parameters that characterise the movement of objects, the so-called movement parameters (MPs) such as speed, acceleration, direction, and derived from the trajectories of objects were taken into account for finding similar trajectories. In [62], sequences of class labels as symbolic representation of MPs for the similarity measure were compared. In this section, we compare our approach to two well-known techniques, namely the Relative Motion (REMO) and the Dynamic Time Warping (DTW).

As a key contribution of this paper, we addressed the applicability of the sequence alignment approach to analyse movements of MPOs. The method is comparable to, for example, REMO and DTW. REMO is an approach that describes motion patterns by changes in the motion attributes of objects such as the speed and motion azimuth of individual MPOs over time [6]. DTW is an algorithm for measuring the similarity between two time series that may vary in time or speed [65]. Unlike traditional distance measures such as the Euclidean distance, DTW can calculate the similarity between two time series that may feature some noise and displacements.

The main difference between our current and previous work is that, at the very basic level, we are investigating the interaction between pairs of MPOs instead of solely looking at the movement of individuals over time. Furthermore, this paper does not investigate the changes in the motion attributes of MPOs. Instead, we examine how the relative changes in the Euclidean distances between MPOs can reveal interesting information.

In our previous work, we compared REMO with DTW and featured some of the advantages and drawbacks of each technique with respect to the same case study used throughout this paper [62]. Although the concept of REMO, DTW and SAMs are uncomplicated and applicable to many research domains, the understanding of these techniques requires some expert knowledge. Different from our previous study, in this paper we demonstrated the usefulness of qualitative information in the analysis and reasoning about movement data. QTC_B_ and QTC_C_ information were cross-pollinated with SAMs.

Unlike DTW, both REMO and the proposed approach in this paper based on SAM can reveal interesting information about motion events retrieved from the interrelation among multiple MPOs. They have this difference that QTC considers the relative motion of one object with respect to another object (i.e., relative movements) and REMO allows the identification and quantification of individual motion behaviour, events of distinct group motion behaviour, so as to relate the motion of individuals to groups [6]. In the DTW approach, we do not investigate movements of multiple objects simultaneously.

The superiority of SAM and DTW over the REMO approach is that REMO is very sensitive to noise, shifts, and distortions in movement data. Thus, drawing analogy between REMO matrices based on such data is challenging. SAM and DTW are less sensitive to noise, shifts, and distortions and give intuitive distance measurements between time series by handling both global and local shifting of the time dimension. Another advantage of DTW and SAM is the ability to handle time series with different lengths, while this is quite challenging with the REMO approach.

From the visual analysis point of view, REMO and SAM support better the human intuition in order to interpret the visual results. Therefore, the high dependency on expert knowledge can be counted as a weakness of the DTW approach.

In order to appraise the robustness of SAMs in the presence of data uncertainty and errors, we give an example of a tango dance in which besides the calibration errors (i.e., errors of positions in the movement data), other sources of noise and errors in capturing movement data with MoCap had a major impact on the recorded data. For example, pairs of dancers performing very close to each other may result in some gaps in tracked data because not all infrared markers attached to the body parts of dancers can be tracked properly.

**Fig 15:**
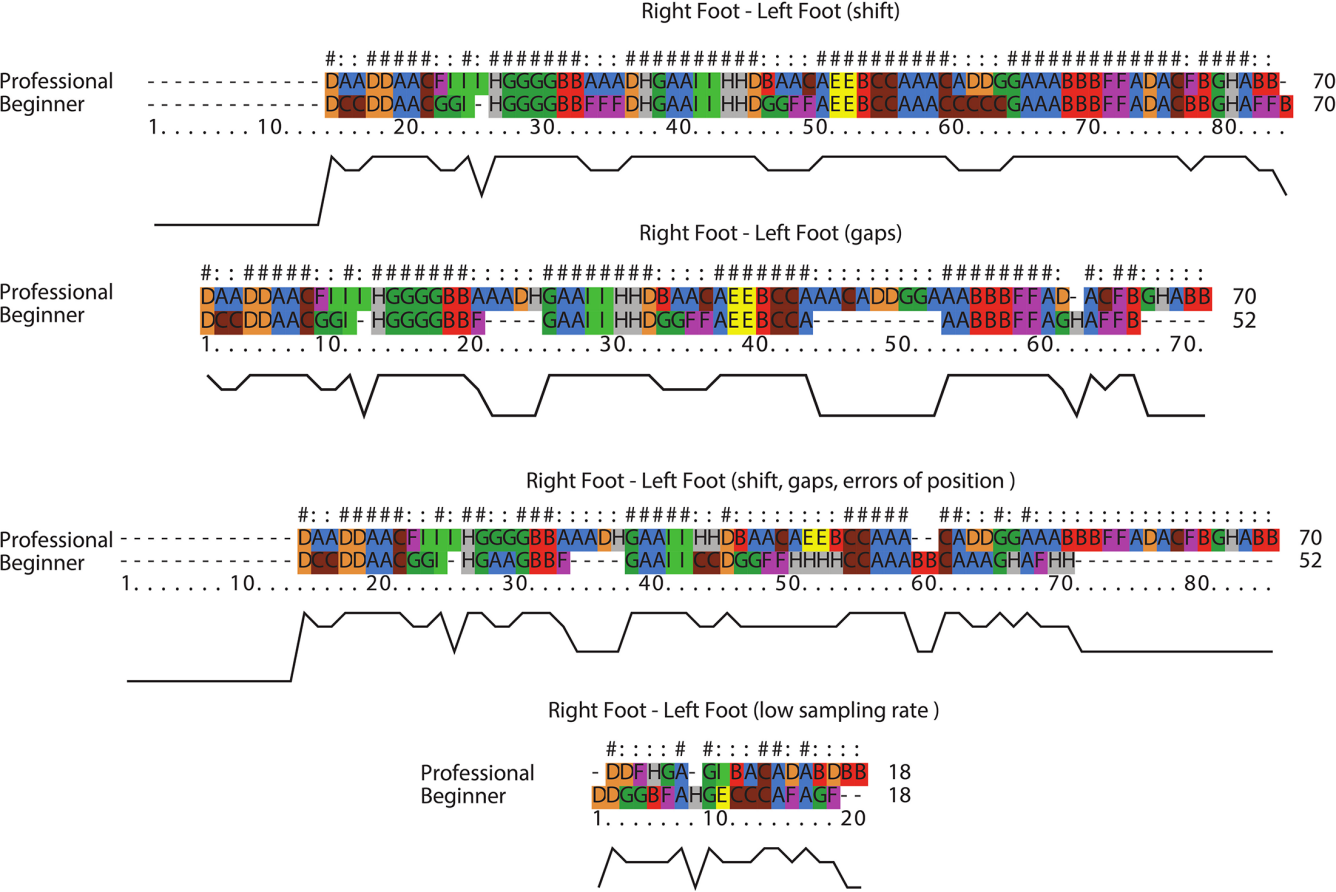
Sample results of QTC_B_ alignment for incomplete data.

The results of global alignment for such data may not be that reliable based on the degree of incompleteness. In Fig 15, we show the results of QTC_B_ aligned sequences for the movements of male partners, both beginner and professional. In Fig 15A, the male partner started his performance with some delays (i.e., shift). From the result of alignment, it can be inferred that SAM is not sensitive to shift.

In Fig 15B, there are some gaps in the QTC_B_ movement sequence of the beginner. These gaps are well recognized in the results of alignment. It should be noted that the location of gaps may change the accuracy of alignment. In Fig 15C, the data includes shifts, gaps, and errors of positions. As might be expected, the more data is incomplete, the worse the alignment is. Compared to samba data, the tango data were not highly repetitive. Therefore, it is worth to know how well the proposed methodology would have performed if the sampling interval was less dense or the movement patterns represented were less regular. In Fig 15D, we show the results of alignment for low sampling rate.

The above visual representations provide complementary insight into the results of alignments. For example, we may judge that the results of SAM are less sensitive to the presence of shifts in sequences (Fig 15A). However, differences due to the gaps in the movements can be clearly observed in more detail from the second representation (Fig 15B). If we look more closely at Fig 15C, we may realise a significant difference in the result due to the combination of shifts, gaps, and errors of position in sequences.

The lower sampling rate has a significant impact on the results of the SAM (Fig 15D). In this regard, detecting movement patterns is quite challenging. All these issues are open research problems and should be comprehensively investigated in the future.

Another remark is that it is not always ideal to align long movement sequences as the results of alignments can be doubtful. Therefore, segmentation of complex time series into smaller units eases perception and learning processes.

## Conclusions and Future Work

Knowledge discovery from moving objects’ trajectories is an important and challenging issue in many research domains. This paper presented a new technique to analyse patterns of relative motion between disjoint MPOs, based on three major steps. In the first step, we described movement of MPOs using the qualitative trajectory calculus (QTC). QTC enables us to express the interactions between moving objects qualitatively. In the next step, a sequence alignment method (SAM) was used to align and assess qualitative movement sequences. Then, in the third step, the results of aligning sequences were presented in the form of dendrograms, in which similar movement sequences were grouped in the same clusters.

The proposed methodology could be used in a wide range of research applications. Movement patterns such as walking, running, jumping, lifting, striking and swimming can be investigated for different purposes. For example, the proposed approach can be used in sports sciences to analyse the movement of athletes with the purpose of rehabilitation, physical education and practice. In this paper, the movements of three samba dancers were analysed to measure the degree of (dis)similarity between the dancers’ movements. Characterising similarity/dissimilarity contributes to a better understanding of how dancers move. The retrieved knowledge can potentially assist dance instructors to examine the movement patterns of novice dancers for educational purposes.

A comprehensive study is beneficial to select suitable compromises between granularity and information. The trajectories captured with the finest time granularity show more details of the movement. It would be worthwhile to investigate the results obtained from different time granularities. On the other hand, the examples presented in this paper were based on relatively short time intervals. In future work, we intend to apply the approach to larger trajectory data sets.

Another avenue for future work will be to enrich the proposed approach by incorporating descriptive statistical analyses. These will provide summaries about QTC movement relations at different time intervals of movement, to bring more insight into the movement patterns and the results of alignments.

## Supporting Information

S1 Dataset The datasets of samba and tango dances used in this paper. (RAR)

S1 Video The videos of samba and tango dances used in this paper. (RAR)
